# Supplementary figures and images for: Proteomic Analysis of Endemic Viral Infections in Neurons offers Insights into Neurodegenerative Diseases
Source: bioRxiv. 2025 Mar 17:2025.03.17.643709. Preprint. [Version 1] doi: 10.1101/2025.03.17.643709 (PMC11957066; doi:10.1101/2025.03.17.643709)

S1

A

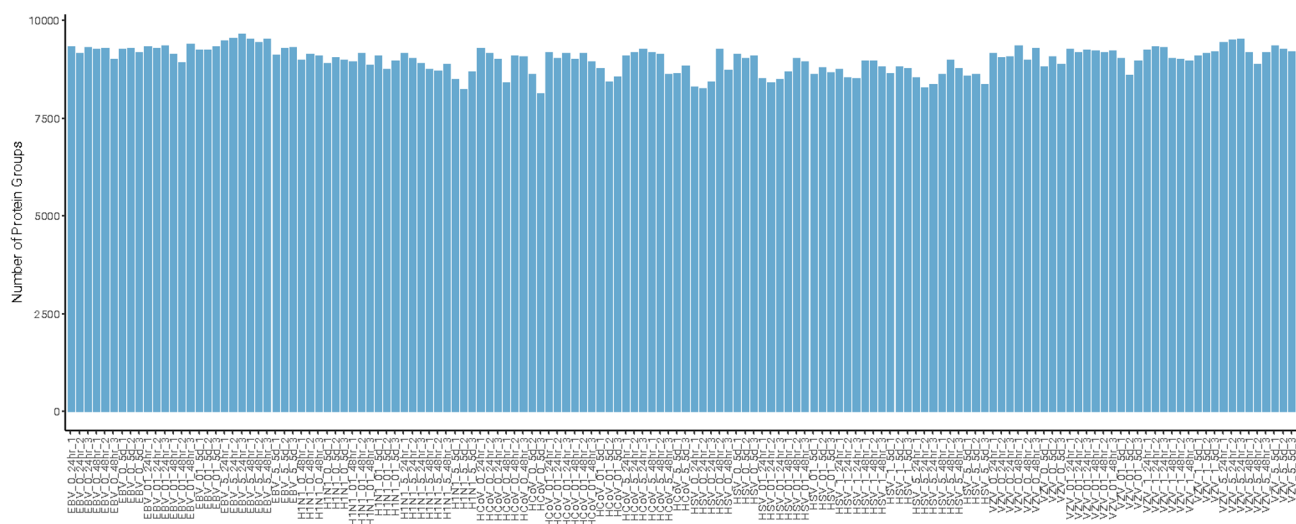

B

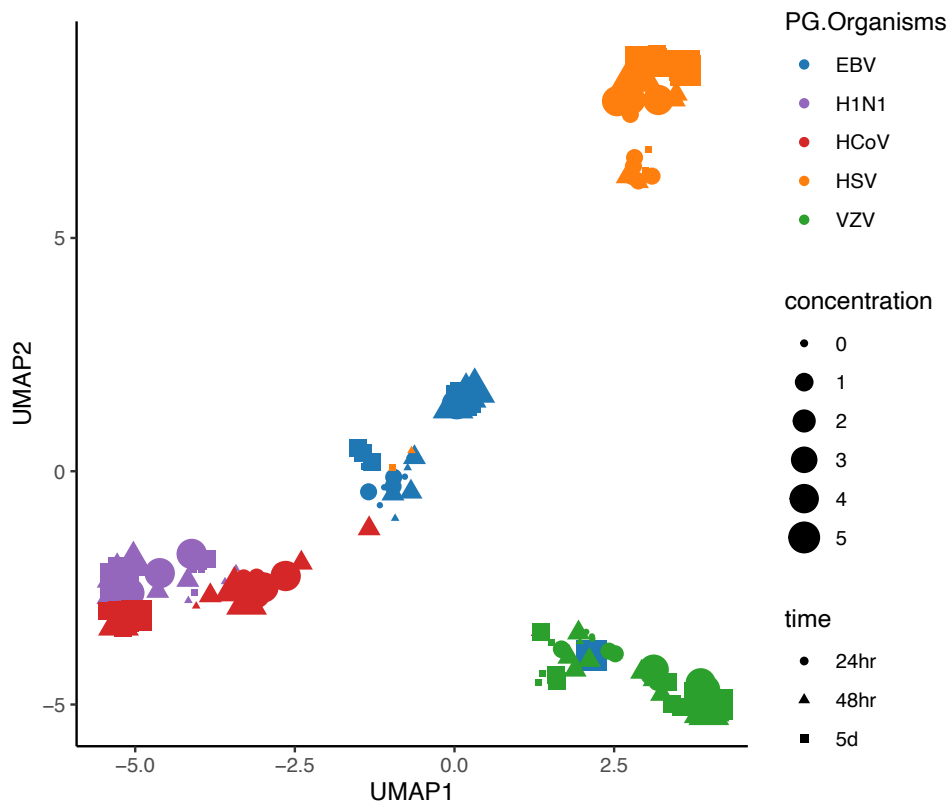

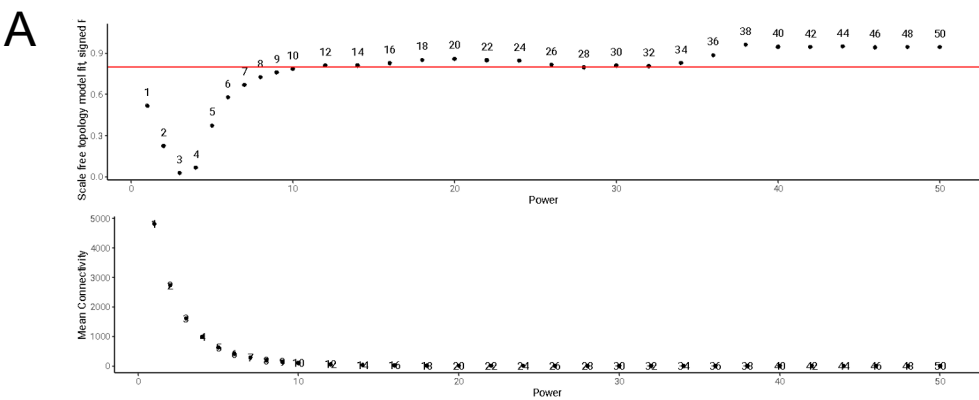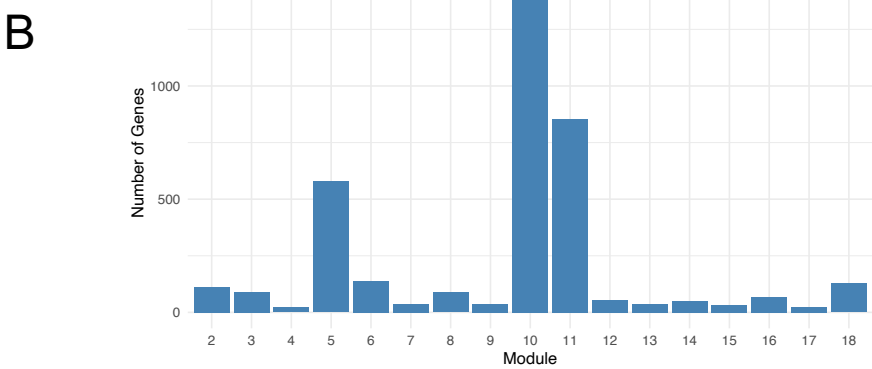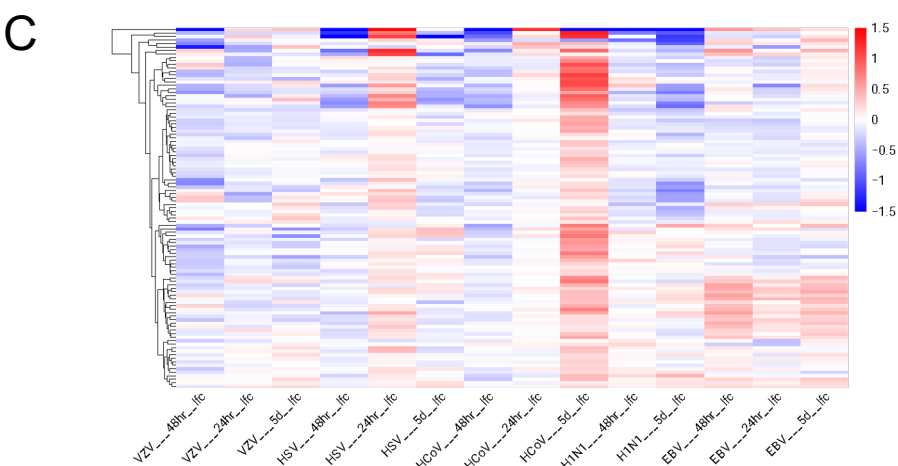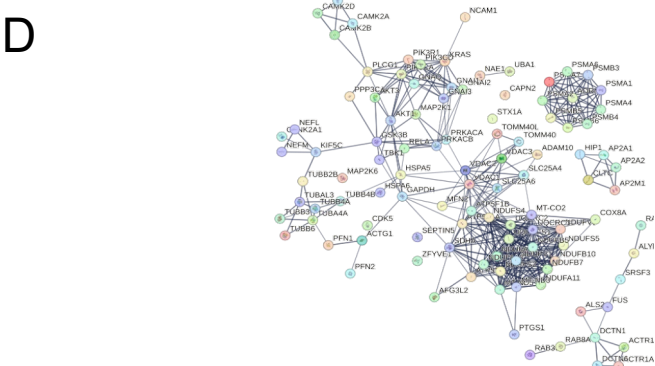

A

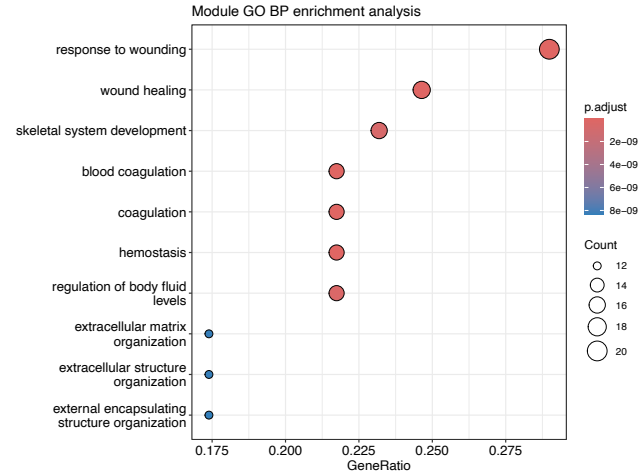

C

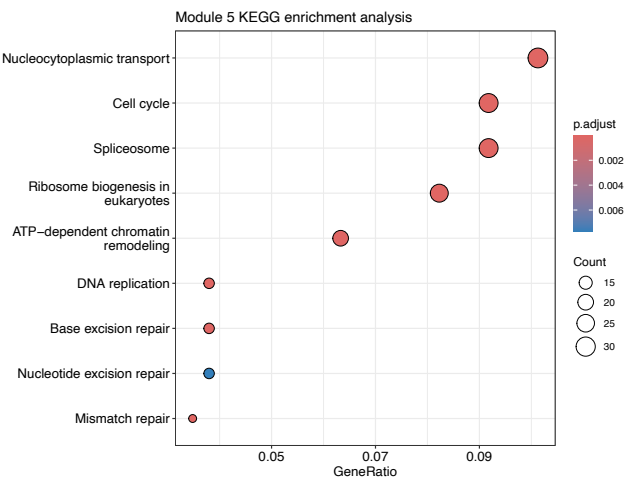

B

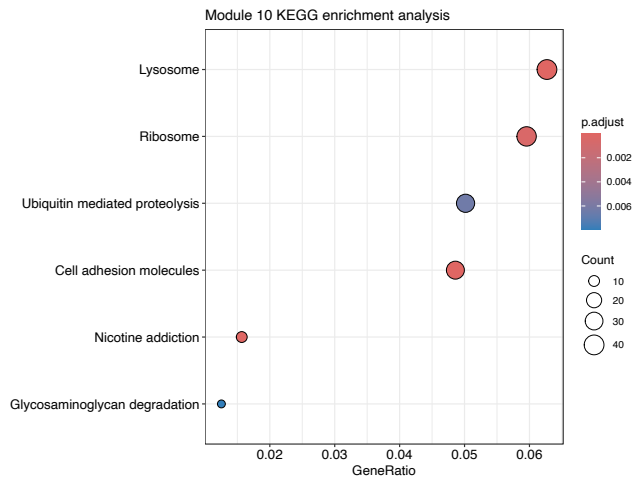

D

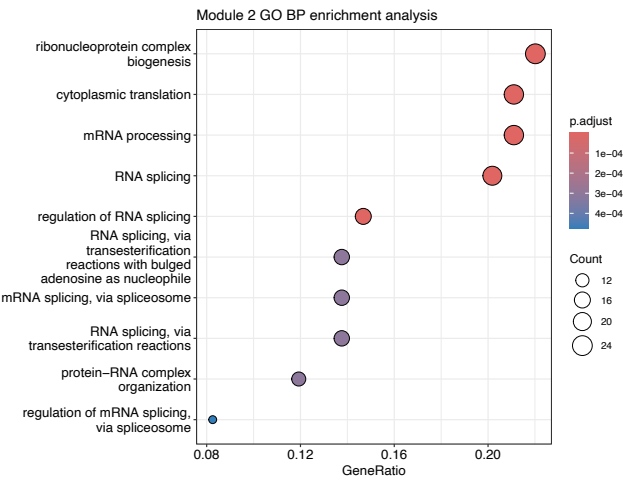

Supplement: Supplement 1 — Supplementary Figure legends Figure S1. Quality Control of the Data (A) Number of human proteins identified across all samples. (B) Uniform Manifold Approximation and Projection (UMAP) analysis showing clustering patterns of proteomic data based on the conditions of viral infection, suggesting the clusters of proteome profile is separated by different viral exposure. Figure S2. WGCNA Data Analysis (A) Plot showing the model fitting by the power soft threshold for network construction in WGCNA. (B) Number of proteins identified in each module. (C) Heatmap showing the log2 FC of neurodegenerative disease (NDD) genes across different viral infection conditions. (D) PPI network of the genes identified in panel C, illustrating the interactions among dysregulated proteins involved in NDDs. Figure S3. Dysregulated Proteins Across All Comparisons (A) Heatmap showing the differential expression of genes across all virus types and time points, highlighting the proteins most significantly dysregulated. (B) UpSet plot identifying genes commonly associated with infection across all viruses, illustrating overlaps in gene dysregulation between the different viral infections. [file media-1.pdf]
